# Spontaneous superficial arteriovenous malformation causing pulsatile tinnitus: A case report

**DOI:** 10.1002/ccr3.3670

**Published:** 2020-12-20

**Authors:** Holt Walters, Sara K. M. Drever, Alaa Abdelgalil, Andrew K. Robson

**Affiliations:** ^1^ Department of Otolaryngology Cumberland Infirmary Carlisle UK; ^2^ Department of Radiology Cumberland Infirmary Carlisle UK

**Keywords:** arteriovenous fistula, neck mass, pulsatile tinnitus, spontaneous

## Abstract

Superficial arteriovenous fistulae in the absence of other complicating features can be easily treatable with simple surgical excision. In this case, the patient's troublesome symptoms were completely cured with a straightforward procedure.

## Graphical abstract

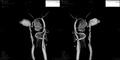

## 1 INTRODUCTION

Spontaneous arteriovenous fistulae are rare in the head and neck region, with <20 cases reported in the medical literature to date. An arteriovenous fistula (AVF) is an abnormal connection between an artery and a vein. Commonly, AVFs are surgically created as a means of vascular access for hemodialysis; however, they have also been reported to occur congenitally, as a result of trauma or iatrogenic injury, and in some cases spontaneously. The authors report an unusual case of a spontaneous AVF between the posterior auricular artery and external jugular vein in a patient presenting with ipsilateral pulsatile tinnitus.

## 2 CASE REPORT

**FIGURE 1:**
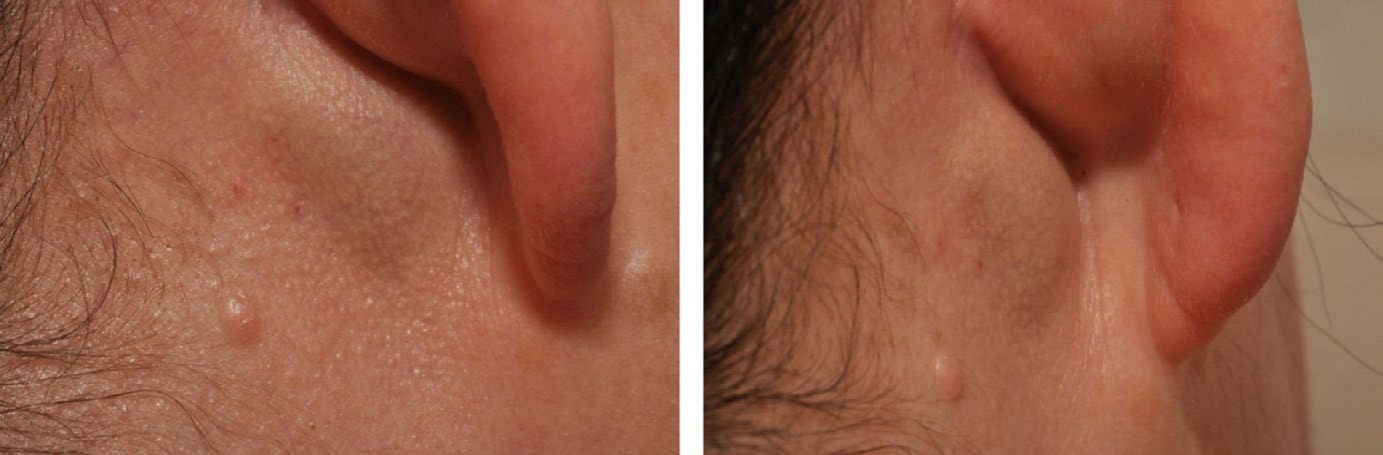
Posterolateral (left) and posterior (right) views of postauricular swelling

A 58‐year‐old lady was referred by her General Practitioner to the Otolaryngology clinic with an 18‐month history of pulsatile tinnitus. This was associated with an enlarging swelling posterior to her right ear. There was no history of trauma, surgery, or percutaneous procedures to her neck, and she was otherwise medically well (Figure 1).

On examination, there was a soft, compressible, pulsatile swelling overlying the right mastoid process that pulsated in synchronicity with her heartbeat. On auscultation, a bruit was audible.

**FIGURE 2:**
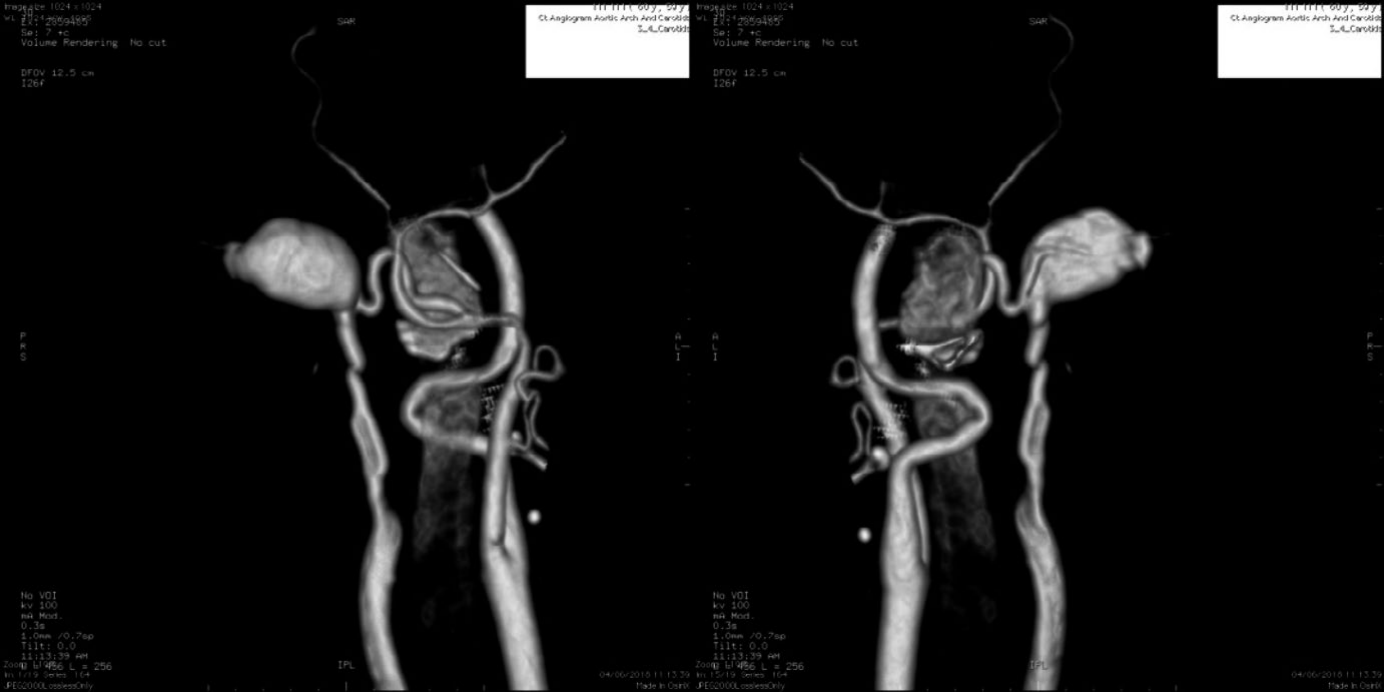
3D reconstructed digital subtraction angiography, anterior (left), and posterior (right) views

Contrast CT displayed evidence of congestion of the posterior auricular division of the right external carotid artery with a large abnormal vascular channel communicating with the external jugular vein. The channel measured approximately 20 × 21 × 13 mm and was located directly below the tip of the right mastoid (Figure 2).

The swelling was excised surgically. Intra‐operatively a large, thin‐walled vein with a small arterial supply was identified deep to the lesion. The lesion was removed in toto. Histology showed cross sections of thick‐walled arterial and thin‐walled veins consistent with a benign vascular lesion.

Following surgical excision, the patient's symptoms completely resolved.

## 3 DISCUSSION

Postauricular arteriovenous fistulae are rare, with only a few cases reported in English language literature to date. Many develop as a result of trauma, spontaneous cases are more commonly associated with genetic or connective tissue diseases such as neurofibromatosis type 1, Marfan's syndrome and Ehler‐Danlos syndrome.^1^ To our knowledge, there have been no reported cases of a spontaneous arteriovenous fistula between the posterior auricular artery and the external jugular vein.

AVFs of the head and neck have been reported to present with a pulsatile tinnitus. Clinical features can also include pulsatile mass, other audiovestibular problems, and neurological symptoms. Untreated they can result in sequelae such as rupture, emboli, and steal syndrome. Larger fistulae can precipitate cardiac failure.^2^

Pulsatile tinnitus is a symptom defined as an auditory perception that is synchronous with the patient's heartbeat. Less than 10% of patients affected by tinnitus will have pulsatile tinnitus.^3^

Causes can be classified as vascular and nonvascular, as listed in Table 1 below.^3^

**Table 1:** Causes of pulsatile tinnitus

Causes of pulsatile tinnitus			
Vascular			
Arterial	Venous	Arteriovenous	Other
Atherosclerosis/Stenosis	High Riding Jugular Bulb	Arteriovenous Fistula	Hyperdynamic Circulatory State
Carotid artery dissection	Dural Venous Sinus Anomalies	Dural Arteriovenous Fistula	Idiopathic Intracranial Hypertension/Pseudotumour Cerebri
Carotid artery aneurysm		Arteriovenous Malformation	

Aberrant internal carotid artery	
Nonvascular	
Osseous	Neoplastic
Paget's disease	Paraganglioma (Glomus)
Otosclerosis	Intracranial Neoplasm
Cavernous Haemangioma	

Most commonly, pulsatile tinnitus as a result of an AVF is due to the abnormality being located along the dura or within a dural sinus. These are referred to as dural AVF. They are also reported occurring as a carotid‐cavernous sinus fistula or a vertebrojugular AVF.^4^

The widely accepted method for radiological evaluation of AVFs is digital subtraction angiography, although CT or MR angiography and Doppler ultrasound are also useful techniques.^5^

Small, asymptomatic AVFs often do not require treatment, although some authors suggest early intervention is appropriate to prevent enlargement of the fistula and avoid any subsequent complications.^1^, ^5^

There are various modalities available for managing AVFs. With the advancement of percutaneous radiologically guided procedures, options include endovascular balloon, stent, or coil embolisation. Alternatively, surgical excision can be undertaken.^2^

## 4 CONCLUSION

Spontaneous AVFs in the head and neck are rare and a significant proportion are associated with genetic or connective tissue diseases. They classically present as a pulsatile neck mass, sometimes accompanied by associated audiovestibular and neurological signs and symptoms. Treatment options include conservative management, endovascular intervention, or surgical excision.

In this case, a simple surgical procedure removed the lesion and cured the patient of her troublesome symptoms.

## CONFLICT OF INTEREST

We received no specific grant from any funding agency, commercial, or not‐for‐profit sectors, and we are not aware of any competing interests.

## AUTHOR CONTRIBUTIONS

HW: main author. SKMD: contributing author. AA: contributed images. AKR: supervised and approved all drafts. We the authors confirm that this report is an original work, and no section of this manuscript has been previously published by or is being considered for publication by other journals. We have written this manuscript collectively with contributions from all authors as listed above. The manuscript has been read and approved by all authors involved. As principal author, I, Holt Walters am willing to take responsibility for the integrity of the content of the manuscript.

## ETHICAL APPROVAL

Approval from an ethics committee was not required in this case; however, verbal and written informed consent was obtained from the patient prior to submission for publication.

## Data Availability

Data sharing is not applicable to this article as the production of this manuscript did not involve the generation or analysis of any datasets.
